# Controlled-Release Fertilizer Improving Paddy Yield and Nitrogen Use Efficiency by Reducing Soil Residual Nitrogen and Leaching Losses in the Yellow River Irrigation Area

**DOI:** 10.3390/plants14030408

**Published:** 2025-01-30

**Authors:** Jingjing He, Ying Wang, Hong Li, Junhua Ma, Xiang Yue, Xiangyu Liang, Yu Hong, Fang Wang, Chenxia Hu, Ruliang Liu

**Affiliations:** 1 School of Agriculture, Ningxia University, Yinchuan 750021, China; hejingjing116@163.com; 2 Institute of Agricultural Resources and Environment, Ningxia Academy of Agro-Forestry Science, Yinchuan 750002, China; wy6789668@163.com (Y.W.); ouxuer333@163.com (Y.H.); berrywang@vip.sina.com (F.W.); 3 Ningxia Agricultural Environmental Protection Monitoring Station, Yinchuan 750002, China; 13995207320@163.com (H.L.); 17795124436@163.com (J.M.); 18202960982@163.com (X.Y.); liangxy0320@163.com (X.L.); 4 National Agricultural Environment Yinchuan Observation and Experiment Station, Ningxia Academy of Agro-Forestry Science, Yinchuan 750002, China; 5 College of Economics and Management, China Jiliang University, Hangzhou 310018, China

**Keywords:** Yellow River irrigation area, paddy yield, N uptake, N use efficiency, N leaching

## Abstract

The unreasonable application of nitrogen (N) fertilizer leads to high nutrient losses and severe potential of agricultural non-point source contamination, which threatens water quality in the upper Yellow River Basin. Therefore, the aim of this study is to explore the effects of N application rates and various control measures on rice yield and N leaching in paddy fields in the Yellow River irrigation area. Four treatments were employed in this study, CK (no N fertilizer application, 0 kg N∙ha^−1^), CRU (controlled-release urea application, 180 kg N∙ha^−1^), OPT (optimal N fertilizer application, 210 kg N∙ha^−1^), and FP (N fertilizer application based on farmer experience, 240 kg N∙ha^−1^), to examine paddy yield, N use efficiency (NUE), N concentrations in leaching water at various soil depths, and N contents along the 0–100 cm depth of the soil profile. The results indicated that the amount of TN leached was 25.14–48.04 kg∙ha^−1^ after different N applications, and the TN leaching coefficients of FP, OPT, and CRU were 10.88%, 11.27%, and 7.07%. Compared to FP and OPT, the CRU significantly reduced the concentrations of TN, ammonium N (NH_4_^+^-N), and nitrate N (NO_3_^−^-N) in the surface and soil water, with average TN leaching decreasing by 31.55% and 27.35% in the years 2022 and 2023, respectively. NO_3_^−^-N was identified as the primary form of N leached from the paddy fields. Compared to FP and OPT treatments, the CRU treatment increased the average paddy yield by 19.99–20.66% and improved the average NUE by 19.04–16.38%. This study revealed that the application of high amounts of N positively affected soil N leaching, and controlled-release urea demonstrates superior efficacy compared to conventional fertilization. The application of controlled-release urea at a rate of 180 kg N∙ha^−1^ not only ensures a good paddy yield but also reduce N losses, which should be recommended to local farmers.

## 1. Introduction

Nitrogen (N) fertilizer plays a key role in enhancing both the yield and quality of paddy crops, with N accounting for over 60% of the total chemical fertilizers applied in paddy cultivation [1,2]. However, the inappropriate application of N fertilizer has resulted in a surplus of N in the soil. A significant proportion of the applied N is not absorbed by crops and is instead lost through processes such as runoff, leaching, and volatilization as ammonia [3]. Research indicated that the N use efficiency (NUE) in China is only 30–35%, which is 15–25 percentage points lower than that observed in developed countries [4,5]. Ammonia volatilization constitutes 68.98–75.27% of N loss in paddy fields, followed by runoff (16.43–23.07%) and leaching (7.67–8.86%) [6]. In single-season paddy fields within the Yangtze River Basin, N runoff losses reached a rate of 10.4 kg∙ha^−1^ [7]. In the Taihu Lake region, ammonia volatilization accounted for approximately 29–35% of the total N applied [8]. When the TN concentration in leaching water exceeds 10 mg∙L^−1^, it can lead to pollution of surface and ground water in paddy fields [9]. In the Ningxia irrigation area, ammonia volatilization represents 45–49% of total N loss, while N leaching accounts for 30–33% [10]. The application of controlled-release N fertilizers has been shown to significantly reduce N loss. Compared to conventional N fertilizers, the NUE increased by 17.7–35.1%, and N runoff loss decreased by 47.8–72.5% when controlled-release N fertilizers were applied [11]. Under the same N application rate, N loss from paddies using controlled-release N fertilizers was lower than that from conventional urea applications [12]. Controlled-release fertilizers not only mitigate environmental N loss but also enhance paddy yield and apparent N recovery [13].

The Ningxia Yellow River irrigation area is one of the four oldest irrigation regions in China and serves as a significant grain production base [14]. Historically, paddy has been a representative crop in this region. Characterized by a typical mid-temperate arid climate, over 85% of agricultural water in this area is sourced from the Yellow River [15,16]. The extensive use of water and fertilizers in paddy cultivation has resulted in severe N leaching during periods of farmland water withdrawal. Consequently, it is crucial to mitigate N input through the application of controlled-release N fertilizers and optimal N application practices to prevent and control non-point source contamination in the Yellow River irrigation area. Total N loss in this irrigation area was 887.51 t, to which maize, wheat, and paddy contributed 25%, 8%, and 67%, respectively, and there was an average N loss coefficient of 1.99% [17]. The total N emissions in the basin account for 52%, with nitrate N comprising 34% and ammonia N 12%. This indicates that non-point source contamination resulting from the excessive application of N fertilizers in the basin remains a significant issue [18]. Therefore, the aims of this study are to (1) explore the effects of optimal N application and controlled-release N fertilizer on N uptake and leaching loss in paddies within the Yellow River irrigation area and to (2) determine reasonable N regulation strategies and provide a basis for the efficient utilization of N fertilizers while controlling non-point source contamination in the Ningxia Yellow River irrigation area.

## 2. Results

### 2.1. Yield of Paddy

The N application rate of N is the primary factor influencing paddy yield. Results from a two-year experiment (Table 1) demonstrated a significant improvement in paddy yield with the application of N fertilizer, with yields increasing progressively alongside higher N application rates across the treatments. Data from 2022 indicated that paddy yield did not increase significantly when the N application rate exceeded 210 kg∙ha^−1^, suggesting that excessive N fertilizer application does not result in a sustained yield increase. Compared to the CK treatment, the average paddy yield in the FP treatment over the two years increased by 2963.85 kg∙ha^−1^, reflecting an impressive yield increase rate of 87.86%. The average paddy yield in the OPT treatment over the two years was 6301.92 kg∙ha^−1^, which was not significantly different from that in conventional N fertilizer application. This indicates that optimal N fertilizer application can maintain yield levels while reducing the overall N application rate. Notably, the average paddy yield in the CRU treatment was the highest, reaching 7604.02 kg∙ha^−1^, which was 20.66% and 19.99% higher than that in the optimal N fertilizer and conventional N fertilizer treatments, respectively. This finding underscores that the application of controlled-release urea can significantly enhance paddy yield.

### 2.2. Nitrogen Uptake and N Use Efficiency of Paddy

Nitrogen use efficiency is a crucial metric for assessing N uptake and utilization in paddy fields. As illustrated in Table 2, total N uptake in each N treatment significantly exceeded that of the CK. In 2022 and 2023, total N uptake in each N treatment increased by 64.12–104.93% and 68.50–110.02%, respectively, compared to CK. Among the various fertilization treatments, the controlled-release urea (CRU) treatment exhibited the highest total N uptake and NUE, following the order: CRU > OPT > FP. The average total N uptake for the FP and OPT treatments over the two years was 104.19 kg∙ha^−1^ and 104.46 kg∙ha^−1^, respectively, with average N use efficiencies of 17.71% and 20.37%. This indicates that the total N uptake and NUE for optimal N fertilizer application were not significantly lower than those for conventional N fertilizer application. Compared to the FP treatment, total N uptake in the CRU treatment increased by 21.08% and 24.64% in 2022 and 2023, respectively, while NUE improved by 19.9% and 18.17%. These results demonstrate that the application of controlled-release urea significantly enhances N absorption and utilization in paddy fields, thereby improving NUE. Furthermore, both NAE and NPP also exhibited improvements.

### 2.3. Nitrogen Concentration in the Surface and Leaching Water

**Figure 1:**
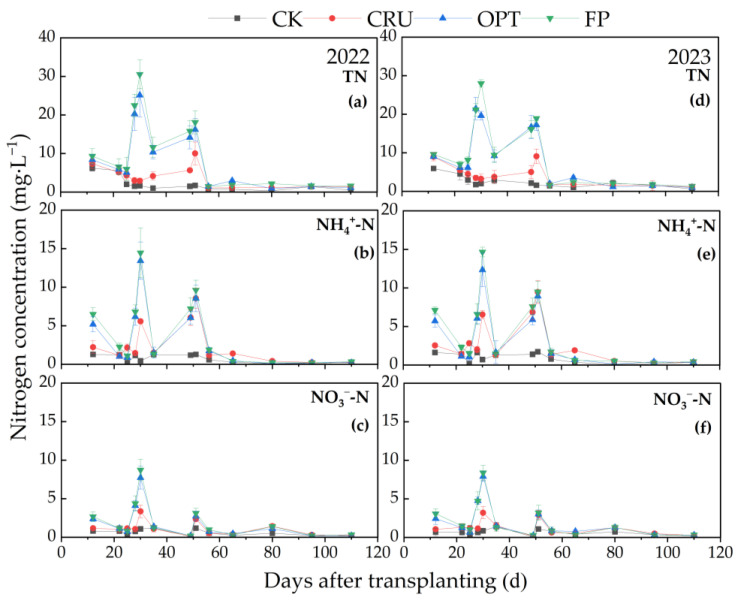
Trends in the TN, NH_4_^+^-N, and NO_3_^−^-N concentrations of surface water in paddy fields under different N fertilizer treatments for two years. Dynamics of TN (**a**), NH_4_^+^-N (**b**), and NO_3_^−^-N (**c**) concentrations of surface water in 2022; dynamics of TN (**d**), NH_4_^+^-N (**e**), and NO_3_^−^-N (**f**) concentrations of surface water in 2023.

The TN concentration in the leaching water at the various soil depths increased progressively with higher N application rates. As illustrated in Figure 1, the peak TN concentrations in the surface water for the FP and OPT treatments were 29.27 mg·L^−1^ and 22.39 mg·L^−1^, respectively, both of which were significantly greater than those observed the CRU treatment. Following topdressing at the tillering stage, the TN concentration in the surface water for the FP and OPT treatments peaked approximately 30 days after sowing and subsequently declined, reaching a low level five days post-peak. In contrast, the TN concentration in the CRU treatment reached a peak value approximately 51 days after sowing, at only 9.55 mg·L^−1^, which was 67.36% lower than the peak in the FP treatment. In line with the TN concentration dynamics, the peak concentrations of NH_4_^+^-N and NO_3_^−^-N in the CRU treatment were only 9.01 mg·L^−1^ and 3.27 mg·L^−1^, respectively. Conversely, the peak NH_4_^+^-N concentrations in the FP and OPT treatments were 14.55 mg·L^−1^ and 12.88 mg·L^−1^, respectively, while the peak NO_3_^−^-N concentrations were 8.56 mg·L^−1^ and 7.81 mg·L^−1^, respectively.

**Figure 2:**
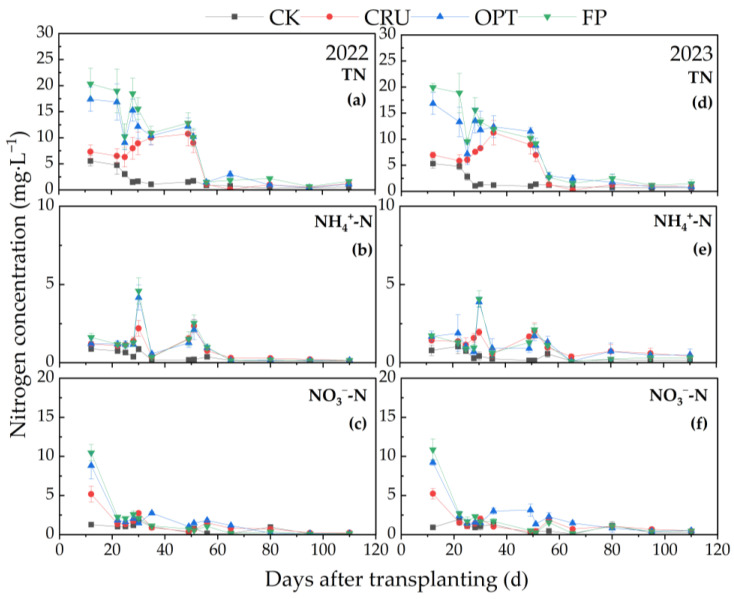
Trends in the TN, NH_4_^+^-N, and NO_3_^−^-N concentrations of the leaching water at the 20 cm soil depth in paddy fields under different N fertilizer treatments for two years. Dynamics of TN (**a**), NH_4_^+^-N (**b**), and NO_3_^−^-N (**c**) concentrations of the leaching water at the 20 cm soil depth in 2022; dynamics of TN (**d**), NH_4_^+^-N (**e**), and NO_3_^−^-N (**f**) concentrations of surface water in 2023.

As illustrated in Figure 2, the trends in TN concentration in the leaching water at a soil depth of 20 cm were comparable to those observed in the surface water. The peak TN concentration for each N treatment occurred between the 12th and 30th days after sowing, followed by a rapid decline to a lower level by the 56th day. Notably, the NH_4_^+^-N concentration in the leaching water at 20 cm significantly decreased, with peak values for each N treatment (0.89–4.33 mg·L^−1^) being 38.62–54.66% lower than those in the surface water (1.45–9.55 mg·L^−1^). In contrast, NO_3_^−^-N concentration peaked approximately 10 days after sowing and then gradually declined to a lower level throughout the paddy growth period.

**Figure 3:**
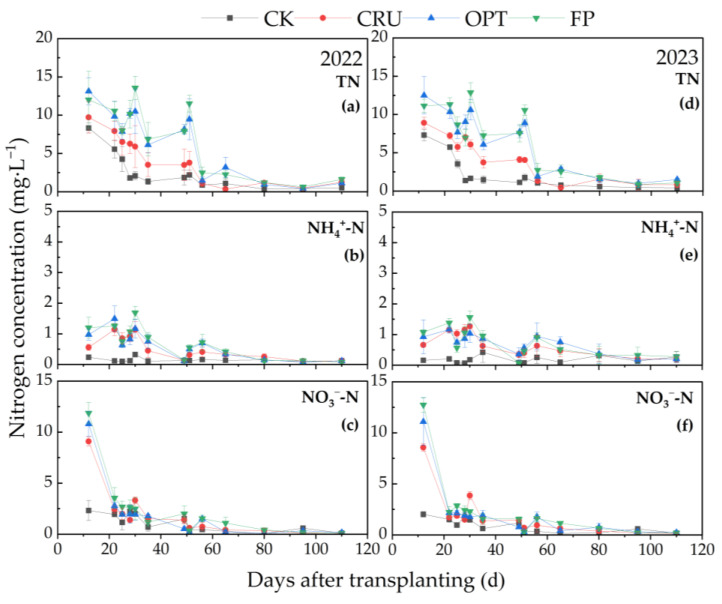
Trends in the TN, NH_4_^+^-N, and NO_3_^−^-N concentrations of the leaching water at the 60 cm soil depth in paddy fields under different N fertilizer treatments for two years. Dynamics of TN (**a**), NH_4_^+^-N (**b**), and NO_3_^−^-N (**c**) concentrations of the leaching water at the 60 cm soil depth in 2022; dynamics of TN (**d**), NH_4_^+^-N (**e**), and NO_3_^−^-N (**f**) concentrations of surface water in 2023.

As shown in Figure 3, the concentrations of N in various forms within the leaching water at a soil depth of 60 cm exhibited a gradual decrease with increasing depth. From the 12th to the 65th day after sowing, the TN concentration in the FP and OPT treatments was higher than that in the CRU treatment and tended to plateau after the 65th day, showing no significant changes thereafter. The peak concentrations of NH_4_^+^-N for each treatment occurred between the 22nd and 30th days post-sowing, with the NH_4_^+^-N concentration in the CRU treatment (1.20 mg·L^−1^) being significantly lower (25.93%) than that in the FP treatment (1.62 mg·L^−1^). Furthermore, the NO_3_^−^-N concentration in the leaching water at 60 cm was comparable to that at 20 cm, rapidly decreasing to a lower level approximately 10 days after sowing.

### 2.4. Amount of N Leached Amount and Leaching Coefficient of Paddy

According to Table 3, the TN leached in the CK treatment was 20.30 kg∙ha^−1^ in 2022 and 12.30 kg∙ha^−1^ in 2023, with the amount in 2023 being significantly lower than that in 2022. Under no N application, the annual leaching of N decreased. In the FP treatment, the amount of TN leached reached 42.41 kg∙ha^−1^, accounting for 17.67% of the N fertilization amount. The TN leached in the OPT and CRU treatments was 39.96 kg∙ha^−1^ and 29.03 kg∙ha^−1^, respectively, representing reductions of 6.13% and 31.55% compared to the FP treatment, thereby significantly decreasing N leaching loss. Over the two years, the amounts of NH_4_^+^-N leached in the OPT treatment were 2.10 kg∙ha^−1^ and 2.23 kg∙ha^−1^, corresponding to reductions of 39.83% and 31.80% compared to the FP treatment. In the CRU treatment, the NO_3_^−^-N leaching amounts over the two years were 17.05 kg∙ha^−1^ and 14.98 kg∙ha^−1^, which were 43.37% and 45.59% lower than those in the FP treatment. The results of the two-year experiment indicated that the average TN leaching coefficients were 10.88% and 11.27% for the FP and OPT treatments, respectively, while the CRU treatment had a significantly lower coefficient of only 7.07%.

NO_3_^−^-N was the predominant form of N leached from the paddy fields, accounting for 25.13–57.29% of TN leaching, whereas NH_4_^+^-N leaching losses represented only 4.77–8.99% of the TN. In 2022, no significant differences were observed in the TN leaching coefficient among the various N treatments. In comparison to the CK treatment, the quantity of N leached in different forms increased with higher N application rates, with the FP treatment resulting in greater leaching than the other treatments. Over two consecutive years, the CRU treatment consistently reduced TN leaching by 25.22–31.63%, NH_4_^+^-N leaching by 15.19–22.94%, and NO_3_^−^-N leaching by 33.4–45.59% when compared to the other N treatments. Therefore, the CRU treatment significantly mitigated N leaching and diminished the risk of agricultural non-point source pollution.

### 2.5. Residual NH_4_^+^-N and NO_3_^−^-N Contents in the Soil Profile

**Figure 4:**
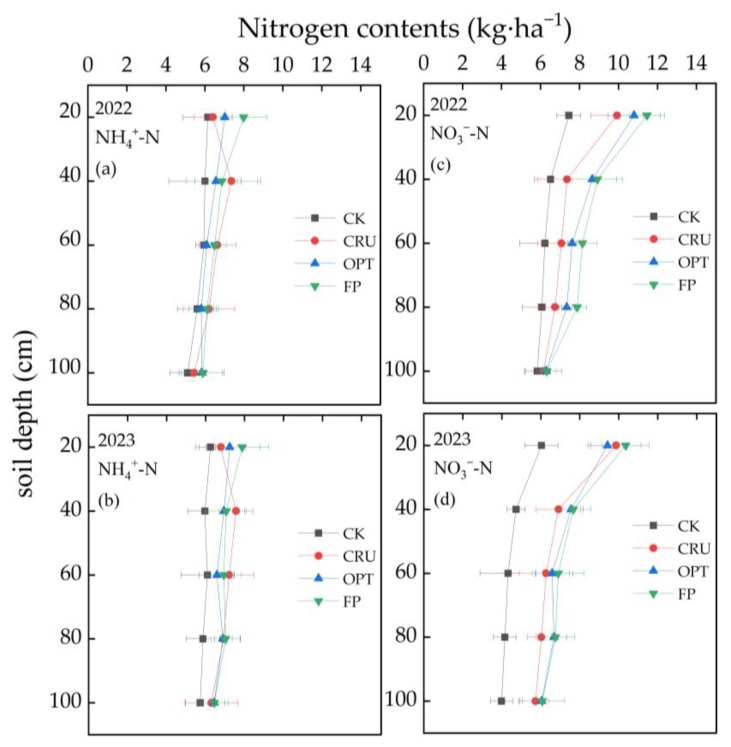
Vertical distributions of NH_4_^+^-N (**a**) and NO_3_^−^-N (**c**) contents in 2022 along the 0~100 cm depth soil profile after paddy harvest. Vertical distributions of NH_4_^+^-N (**b**) and NO_3_^−^-N (**d**) contents in 2023 along the 0~100 cm depth soil profile after paddy harvest. The horizontal bars mean standard deviations of the means CK, CRU, OPT, and FP.

Soil inorganic N is the primary form of N absorbed by crops, and its migration within the soil profile is significantly influenced by the levels of N fertilizer application and the extent of irrigation. According to Figure 4, within the 0–100 cm soil profile, the NH_4_^+^-N content in each treatment remained relatively consistent at various soil depths. In contrast, the NO_3_^−^-N content was markedly higher than the NH_4_^+^-N content and exhibited a clear decline with increasing soil depth. At the 0–20 cm soil depth, the NH_4_^+^-N and NO_3_^−^-N contents in the FP treatment were the highest, measuring 7.93 kg∙ha^−1^ and 10.91 kg∙ha^−1^, respectively, while those in the CK treatment were the lowest, at only 6.20 kg∙ha^−1^ and 6.74 kg∙ha^−1^, respectively. At the 20–40 cm soil depth, the NH_4_^+^-N content in the CK treatment was significantly lower than that in the other N fertilizer treatments. The order of NO_3_^−^-N content was observed as FP > OPT > CRU > CK. At the 60–100 cm soil depth, there were no significant differences in NH_4_^+^-N content among the treatments; however, the NO_3_^−^-N content decreased gradually with increasing soil depth.

During the two-year study, the highest NO_3_^−^-N content was observed at the 0–20 cm soil depth. Over the growing period, the NO_3_^−^-N content in the CK treatment gradually decreased with increasing soil depth, while the NO_3_^−^-N content in the CRU treatment was lower than that in the FP treatment. At the 20–40 cm soil depth, the NO_3_^−^-N content in the CRU treatment was 7.13 kg∙ha^−1^, which was 13.99% and 8.59% lower than that in the FP and OPT treatments, respectively, indicating a reduction in the leaching of NO_3_^−^-N into deeper soil layers. At the 0–20 cm soil depth, the NH_4_^+^-N content in the CRU treatment was 6.60 kg∙ha^−1^, which was 16.77% and 7.43% lower than that in the FP and OPT treatments, respectively. Compared to conventional N fertilizer application, the residual NH_4_^+^-N and NO_3_^−^-N contents along the soil profile decreased by 6.88% and 4.54%, respectively, under controlled-release urea application and optimal N fertilizer application, thereby reducing the risk of N leaching loss.

### 2.6. Relationships Between N Leaching and the Physical–Chemical Properties of Soil

**Figure 5:**
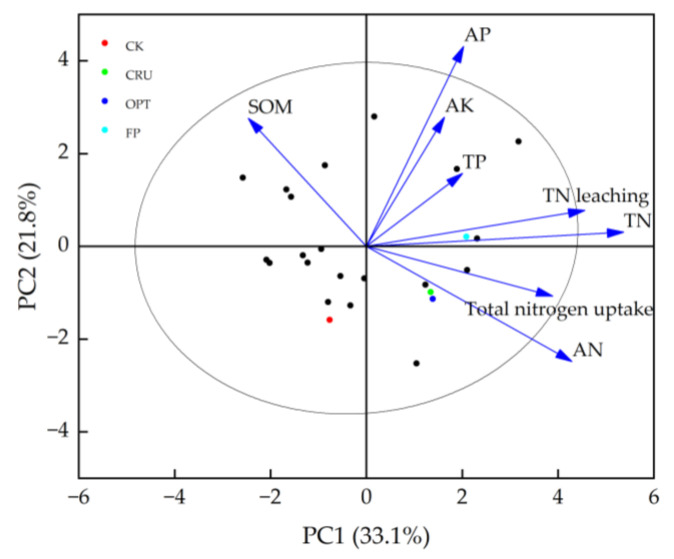
Principal component analysis (PCA1, PCA2) showing trait vectors (soil organic matter, available phosphorus, available potassium, available N, total N, total N leaching, total N uptake) of N leaching and physical–chemical properties of the soil.

The overall effects of N application at varying rates on N leaching and soil factors were analyzed using principal component analysis (PCA). The first two axes accounted for 54.9% of the variation in TN leaching and soil factors, indicating that these factors significantly influenced N uptake and N leaching. The PCA results (Figure 5) demonstrated a strong correlation between TN content in the soil and TN leaching, suggesting that TN is the primary factor affecting N leaching. Furthermore, this study found that TN leaching increased with higher N application rates. Additionally, available N, total phosphorus, available potassium, and available phosphorus all exhibited positive correlations with TN leaching.

## 3. Discussion

### 3.1. Effects of Optimal N Application on Paddy Yield

N fertilizer plays a vital role in paddy production. Appropriate application of N fertilizer can enhance the uptake and utilization of N by crops, ensuring both high quality and high yield [19,20]. However, excessive N fertilizer application not only reduces paddy yields but also leads to potential risk of non-point source contamination [21]. Controlled-release urea has been shown to effectively reduce N loss and mitigate non-point source contamination. The findings of this study indicate that, compared to the CRU treatment, the FP treatment did not result in increased paddy yield despite a 33.33% increase in the N application rate, corroborating the findings of Dong et al. [22]. A single application of controlled-release N fertilizer during the growing season significantly enhanced the availability of N in the soil, improved paddy growth, and resulted in higher paddy yields with reduced N application, as reported by Yang et al. [23]. In this study, the paddy yield under the controlled-release N fertilizer treatment was significantly higher than that under optimal and conventional fertilization methods, demonstrating that controlled-release N fertilizer can effectively satisfy the N nutritional requirements of paddy plants throughout the cultivation period, thereby promoting growth and increasing yields.

### 3.2. Effects of Optimal N Application on N Leaching Loss

The long-term and large-scale application of N fertilizer results in the accumulation of N in the soil from year to year, leading to the leaching of significant amounts of N in various forms. Reducing N application can effectively decrease N leaching [24]. NO_3_^−^-N is the primary form of N leached from paddy fields. This is because the existence of a large number of nitrifying bacteria leads to extensive nitrification in soil. Research conducted by Chen et al. revealed that NH_4_^+^-N and NO_3_^−^-N were the main components of TN in both surface water and leaching water, accounting for 77.1% and 83.6% of the TN, respectively [25]. As soil depth increases, the concentration of NH_4_^+^-N in the leaching water clearly decreases, as NH_4_^+^-N typically remains in the upper soil layers where it is readily absorbed by paddy plants and soil particles. In contrast, NO_3_^−^-N contributes to nitrate pollution through the process of nitrification [26,27]. Compared to conventional fertilization methods, the application of controlled-release N fertilizer reduces N output from paddy fields to the environment by 36.3 kg ha^−1^ [6]. The peak of N leaching occurs during the initial stage of fertilization, which is a critical period for controlling N loss. The coating applied to controlled-release N fertilizer effectively prevents the rapid decomposition of N and facilitates a gradual release of nutrients in accordance with the N demands of paddy plants at various growth stages. This approach enhances N absorption and utilization while reducing the risk of N leaching loss [28].

The TN concentration peaked during the early stages of paddy growth in both years before declining rapidly. N leaching in paddy fields primarily occurs during this early growth phase due to the presence of residual N in the soil, the application of a substantial base fertilizer, and significant initial drainage, according to Cao et al. [29,30]. In comparison to other N fertilizer treatments, the leached N concentrations and TN leaching in the FP treatment were notably higher. Optimal N fertilization can minimize fertilizer waste and N loss while ensuring good paddy yield and NUE [31]. In this study, when conventional urea was utilized as the N source, the average leaching coefficients of TN in the FP and OPT treatments were 10.88% and 11.27%, respectively, whereas the average leaching coefficient of TN in the CRU treatment was only 7.07%, aligning with the findings of previous research [23]. The N concentrations in both surface water and leaching water in the OPT treatment were significantly lower than those in the FP treatment. Additionally, the peak N concentration in the surface water and leaching water of the CRU treatment was markedly lower than that of the other treatments, and the peak occurred later. Compared to FP and OPT, the amount of TN leached from the controlled-release N fertilizer decreased by 31.55% and 27.35%, respectively, corroborating the results of Peng et al. [32]. Consequently, reducing N input and employing controlled-release N fertilizer can effectively decrease N loss in paddy fields.

In addition, PCA showed the correlation between soil nutrients and TN leaching. Organic acids and other substances produced by organic matter decomposition can promote the release of insoluble nitrogen in soil. A proper amount of phosphorus can promote the development of the rice root system, but excessive accumulation of phosphorus can change soil physical and chemical properties, affect soil structures, and change soil permeability [33,34]. When the K^+^ concentration is high, the ion exchange between K^+^ and NH_4_^+^ leads to an increase in NH_4_^+^ concentration in soil and thus leaching with water.

### 3.3. Effects of Optimal N Application on N Use Efficiency

Currently, the NUE of paddy in China is only 30–35%. Excessive N application results in increased residues of ammonium N and nitrate N in the surface soil of paddy fields. In comparison to FP, the NUE under optimal fertilization improved by an average of 16.07% over two years, while the residual ammonium N and nitrate N decreased by 4.80% and 4.35%, respectively. Additionally, controlled-release urea has been shown to enhance NUE by more than 10 percentage points compared to conventional urea, subsequently increasing the NAE by 24.97–54.02%, as reported by Chen Kun et al. [35]. This study found that NUE was highest with controlled-release N fertilizer, which increased by an average of 19.04 percentage points compared to FP. Controlled-release fertilizer, which caters to the N nutritional requirements of paddy at various growth stages, can significantly improve NUE, according to Sun Ting et al. [36,37]. In this study, the two-year average NUE was found to be 36.74%, with an NAE of 23.5 kg∙kg^−1^ and NPP of 42.24 kg∙kg^−1^ in the CRU treatment. These findings align with the results reported by Dobermann et al. [38]. Compared to conventional N fertilizer application, the use of controlled-release N fertilizers significantly enhances aboveground N accumulation in paddy plants, with NUE increasing by 17.10–34.11%, as reported by Li Yunchun et al. [39]. This improvement can be attributed to the slower N release rate of controlled-release urea compared to conventional urea, which enables vegetative organs, such as the stems and leaves of paddy plants, to grow more vigorously and absorb more N. Following the booting stage, the increased production of photosynthetic substances can be effectively transferred to paddy grains, further enhancing NUE [40]. Therefore, optimizing N application practices and utilizing controlled-release N fertilizers can promote N uptake and improve NUE.

## 4. Materials and Methods

### 4.1. Experimental Site

The experiment commenced in 2020, utilizing experimental data collected from 2022 to 2023. The experimental site is located in Wanghong Town, Yongning County, which serves as the experimental station of the Ningxia Academy of Agriculture and Forestry Sciences (106°12′55″ E, 38°13′3″ N). This site is situated within the critical zone of the Ningxia Yellow River irrigation area, characterized by a typical mid-temperate arid climate. The average annual sunshine duration ranges from 2868 h to 3060 h, while the average annual temperature varies between 8.5 °C and 9.2 °C. Annual precipitation averages between 180 mm and 200 mm, and annual pan evaporation is estimated at 1600 mm to 2000 mm. The value of pH is 8.21 in 0–20 cm soil depth. The basic soil properties at a depth of 0–100 cm prior to the experiment are detailed in Table 4, indicating a moderate level of soil fertility. The growth periods of the tested paddy spanned from 9 May 2022, to 17 October 2022, and from 4 May 2023, to 8 October 2023. The rice of Fuyuan 4 was used as the test material in this study.

### 4.2. Experimental Design

Four treatments were employed in this experiment: CK (no N fertilizer application, 0 kg∙ha^−1^), CRU (controlled-release urea application, 180 kg∙ha^−1^), OPT (optimal N fertilizer application, 210 kg∙ha^−1^), and FP (N fertilizer application based on farmer experience, 240 kg∙ha^−1^). Three replicate plots were set for each treatment and randomly arranged in blocks, with each plot covering an area of 60 m^2^ (6 m × 10 m). The same amounts of phosphate fertilizer (P_2_O_5_, 90 kg∙ha^−1^) and potassium fertilizer (K_2_O, 45 kg∙ha^−1^) were utilized across all treatments, applied once as base fertilizers.

**Figure 6:**
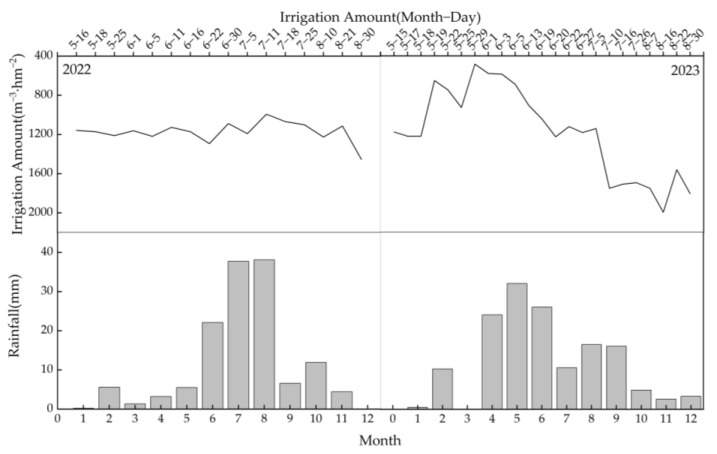
Irrigation and precipitation during the paddy growth period. The amount of irrigation water applied was determined via an electromagnetic flowmeter, and the precipitation data were obtained from the China Meteorological Network.

The controlled-release urea was provided by Shandong Yantai Agricultural Capital Sun Fertilizer Company (Yantai, Shandong Province, China), containing a total N content of 44%. Meanwhile, the conventional urea was employed for the OPT and FP treatments, and it was provided by Sinopec Group (China Petrochemical Corporation, Beijing, China), containing a total N content of 46%. All phosphate and potassium fertilizers were applied once before sowing as base fertilizers, specifically superphosphate (P_2_O_5_ content of 46%) for phosphate and potassium chloride (K_2_O content of 60%) for potassium. In the CRU treatment, polymer-coated urea was utilized as the N source, while conventional urea was employed for the OPT and FP treatments. The polymer-coated urea was applied once as a base fertilizer in the CRU treatment, whereas in the OPT and FP treatments, 60%, 20%, and 20% of the conventional urea were applied before sowing, during the tillering stage, and during the booting stage, respectively. Irrigation schedules were determined based on the arrival date of water in the Yellow River irrigation area and the water requirements of the paddies. Figure 6 illustrates the specific irrigation and precipitation amounts during the paddy growth period.

### 4.3. Sampling and Measurements

Plant sample analysis: At the time of harvest, paddy plant samples were collected from 1 m of each plot. After 20 min in an oven at 70 °C, the samples were dried at 105 °C to a constant weight; the dried materials were used to determine the total N content of both the paddy grain and straw, which were digested with H_2_SO_4_-H_2_O_2_ and analyzed with an automatic semi-micro Kjeldahl N analyzer.

Collection and analysis of leaching water: A sampling device for leaching water was installed in each plot, designed by our team and protected by intellectual property rights [41]. This device facilitated the collection of soil water samples from depths of 20 cm and 60 cm. Concurrently, field surface water samples were randomly collected at three points and mixed with a syringe. These samples were later analyzed in the laboratory for total N (TN), ammonium N (NH_4_^+^-N), and nitrate N (NO_3_^−^-N) contents. The total N content was determined by potassium persulfate oxidation–ultraviolet spectrophotometry, while ammonium N and nitrate N contents were determined by flow injection analysis.

Soil samples were collected from depths of 0–20 cm, 20–40 cm, 40–60 cm, 60–80 cm, and 80–100 cm during the harvest stage. Soil samples were also obtained from 3 points within each plot and combined. Fresh soil samples were frozen in sealed bags and extracted with KCl (1 mol), after which the contents of nitrate N and ammonium N in the soil were determined by flow injection analysis. Meanwhile, total phosphorus was determined by the alkali fusion-Mo-Sb Anti spectrophotometric method and available phosphorus was determined by the Sodium hydrogen carbonate solution-Mo-Sb Anti spectrophotometric method [42].

### 4.4. Calculations

The N agronomic efficiency (*NAE*) was calculated with the following equation [43]:

*NAE* (kg·kg^−1^) = (*Y_x_* − *Y*_0_)/*N_f_*, (1)

where *Y_x_* is the paddy grain yield in N fertilizer application treatments (kg∙ha^−1^), *Y*_0_ is the yield with no N fertilizer (kg∙ha^−1^), and *N_f_* is the amount of N fertilizer input (kg∙ha^−1^).

N partial productivity (*NPP*) was calculated with the following equation [44]:

*NPP* (kg·kg^−1^) = *Y_x_*/*Nf*, (2)

where *Y_x_* is the paddy grain yield in N fertilizer application treatments (kg∙ha^−1^) and *N_f_* is the amount of N fertilizer input (kg∙ha^−1^).

N use efficiency (*NUE*) was calculated with the following equation [45]:

*NUE* (%) = (*N_t_* − *N_c_*)/*Nf* × 100, (3)

where *N_t_* is the total N uptake in the fertilized plot (kg∙ha^−1^), *N_c_* is the CK plot (kg∙ha^−1^), and *N_f_* is the amount of N fertilizer input (kg∙ha^−1^).

N leaching loss (*P*) was calculated using the following equation:

(4) $$P=\sum_{i=1}^{n}Ci\times Vi$$

where *Ci* is the concentration of N in the ith leached water (mg·L^−1^), and *Vi* is the volume of leached water at the *i*th time (L).

### 4.5. Statistical Analysis

Microsoft Excel 2016 and Origin 2024b were used for principal component analysis (PCA) and one-way analysis of variance (ANOVA). The least significant difference (LSD) test was used to compare the means with a 5% threshold of significance.

## 5. Conclusions

Compared to other N application treatments, CRU significantly reduced N concentration and N leaching in both surface water and leaching water. The results from the PCA indicated that total N was the most critical factor influencing TN leaching. The CRU treatment also led to a notable increase in yield compared to the FP and OPT treatments. Furthermore, N uptake and NUE in the CRU treatment were significantly higher than those in the other treatments. Specifically, NUE in the CRU treatment increased by 19.04% and 16.38% compared to the FP and OPT treatments, respectively, while NAE increased by 11.01 kg∙kg^−1^ and 9.56 kg∙kg^−1^, respectively. No significant difference was found between the FP and OPT treatments. Notably, the average yield in the OPT treatment decreased by 35.33 kg∙ha^−1^ compared to the FP treatment. Although the OPT treatment positively impacted the farmland environment, the N application rate was deemed unsuitable. To enhance paddy yield, improve NUE, and minimize N loss, the application of controlled-release urea (180 kg∙ha^−1^) is recommended as the optimal treatment. This study accurately quantified the effects of various N application rates on soil N balance and revealed the quantitative relationships between N application rates and the transformation, accumulation, and loss of soil N. Additionally, the primary factors influencing TN leaching were clarified. The findings of this study are crucial for advancing N fertilizer management and establishing appropriate fertilization strategies in paddy cultivation in northwest China. In addition, attention should be given to the interplay between irrigation and fertilization levels to achieve a better balance in N fertilizer management for paddy fields.

## Figures and Tables

**Table 1:** Paddy yield under different N fertilizer treatments after harvest.

Treatment	Yield in 2022 (t∙ha^−1^)	Yield in 2023 (t∙ha^−1^)	Average Yield (t∙ha^−1^)	Ratio of Yield Increase (%)
CK	3.8 ± 0.2 c	3.0 ± 0.5 b	3.4 ± 0.2 c	
CRU	8.5 ± 0.6 a	6.7 ± 0.6 a	7.6 ± 0.2 a	125.4 ± 6.1 a
OPT	6.9 ± 0.6 b	5.7 ± 0.7 a	6.3 ± 0.5 b	86.8 ± 15.5 b
FP	7.0 ± 0.3 b	5.7 ± 0.6 a	6.3 ± 0.4 b	87.9 ± 12.7 b

CK, no N fertilizer application; CRU, controlled-release urea application; OPT, optimal N fertilizer application; FP, N fertilizer application based on farmer experience. The values before and after the “±” indicate the average value and standard deviation, respectively. The letters following the values represent significant differences at *p* < 0.05.

**Table 2:** Nitrogen uptake, N use efficiency (NUE), N agronomic efficiency (NAE), N partial productivity (NPP) under different N fertilizer treatments during the paddy growing season.

Year	Treatment	N Uptake by Paddy Straw (kg∙ha^−1^)	N Uptake by Paddy Grain (kg∙ha^−1^)	Total N Uptake (kg∙ha^−1^)	NUE (%)	NAE (kg∙kg^−1^)	NPP (kg∙kg^−1^)
2022	CK	22.36 ± 6.19 c	45.23 ± 4.82 c	67.60 ± 7.56 c			
	CRU	40.53 ± 4.89 a	98.00 ± 6.97 a	138.53 ± 3.50 a	39.41 ± 1.94 a	26.45 ± 3.40 a	47.36 ± 3.40 a
	OPT	30.66 ± 2.52 ab	80.29 ± 5.59 b	110.95 ± 4.05 b	20.64 ± 1.93 b	14.87 ± 2.72 b	32.80 ± 2.72 b
	FP	32.03 ± 0.73 b	81.38 ± 8.22 b	114.41 ± 7.54 b	19.51 ± 3.14 b	13.45 ± 1.31 b	29.13 ± 1.31 b
2023	CK	20.53 ± 6.06 b	35.23 ± 6.32 b	55.76 ± 11.93 b			
	CRU	34.12 ± 1.82 a	82.99 ± 13.77 a	117.11 ± 15.20 a	34.08 ± 8.45 a	20.56 ± 3.39 a	37.13 ± 3.39 a
	OPT	29.87 ± 0.69 ab	68.10 ± 8.05 a	97.97 ± 7.82 a	20.10 ± 3.72 b	13.02 ± 3.34 b	27.22 ± 3.34 b
	FP	29.01 ± 9.78 ab	64.95 ± 15.53 a	93.96 ± 17.11 a	15.91 ± 7.13 b	11.25 ± 2.29 b	23.68 ± 2.29 b

CK, no N fertilizer application; CRU, controlled-release urea application; OPT, optimal N fertilizer application; FP, N fertilizer application based on farmer experience. The values before and after the “±” indicate the average value and standard deviation, respectively. The letters following the values represent significant differences at *p* < 0.05.

**Table 3:** The amounts of TN, NH_4_^+^-N, and NO_3_^−^-N leaching and their leaching coefficients in paddy fields under different N fertilizer treatments.

Year	Treatment	TN Leaching (kg∙ha^−1^)	TN Leaching Coefficient (%)	NH_4_^+^-N Leaching (kg∙ha^−1^)	NH_4_^+^-N Leaching Coefficient (%)	NO_3_^−^-N Leaching (kg∙ha^−1^)	NO_3_^−^-N Leaching Coefficient (%)
2022	CK	20.30 ± 2.58 e		1.31 ± 0.10 d		8.67 ± 1.54 c	
	CRU	32.91 ± 3.76 cd	7.01 ± 2.09 b	2.96 ± 0.55 bc	0.92 ± 2.25 b	17.05 ± 1.82 b	4.66 ± 2.16 ab
	OPT	44.01 ± 3.76 bc	11.29 ± 1.79 b	2.10 ± 0.09 cd	0.38 ± 2.19 a	25.60 ± 4.18 b	8.06 ± 1.96 b
	FP	48.04 ± 4.17 ab	11.56 ± 1.74 b	3.49 ± 0.44 b	0.91 ± 1.68 ab	30.11 ± 3.84 ab	8.93 ± 1.96 b
2023	CK	12.30 ± 2.11 d		1.09 ± 0.26 c		6.33 ± 1.12 b	
	CRU	25.14 ± 2.22 c	7.13 ± 1.24 b	2.52 ± 0.35 b	0.79 ± 1.09 a	14.98 ± 1.55 bc	4.81 ± 1.46 a
	OPT	35.90 ± 4.81 b	11.24 ± 2.29 a	2.23 ± 1.04 b	0.54 ± 1.83 ab	23.36 ± 3.27 c	8.11 ± 1.11 a
	FP	36.77 ± 2.75 b	10.20 ± 1.15 ab	3.27 ± 0.43 bc	0.91 ± 1.21 a	27.53 ± 2.19 b	8.83 ± 0.94 a

CK, no N fertilizer application; CRU, controlled-release urea application; OPT, optimal N fertilizer application; FP, N fertilizer application based on farmer experience. The values before and after the “±” indicate the average value and standard deviation, respectively. The letters following the values represent significant differences at *p* < 0.05.

**Table 4:** The basic soil properties at 0~100 cm in the study area.

Soil Depth (cm)	Bulk Density (g·cm^−3^)	Total Porosity (%)	Total Salt (g·kg^−1^)	Organic Matter (g·kg^−1^)	Total N (g·kg^−1^)	Available N (mg·kg^−1^)
0~20	1.36	48.7	0.49	10.74	1.01	38.66
20~40	1.36	48.8	0.40	8.71	0.85	26.98
40~60	1.53	42.3	0.39	5.26	0.40	25.12
60~80	1.64	39.0	0.35	4.41	0.31	24.31
80~100	1.44	45.4	0.31	3.15	0.29	23.58

## Data Availability

The original contributions presented in this study are included in the article.
